# Evaluating the impact of a 10 year reduction in critically important antibiotic use on the occurrence of antibiotic resistance in *E. coli* from cattle, dogs and cats in France

**DOI:** 10.1093/jacamr/dlaf207

**Published:** 2025-11-18

**Authors:** Lucie Collineau, Soukaina Ait Ali, Servane Bareille, Claire Chauvin, Marisa Haenni, Nathalie Jarrige, Jean-Yves Madec, Delphine Urban, Géraldine Cazeau

**Affiliations:** Epidemiology and Surveillance Support Unit, University of Lyon—French Agency for Food, Environmental and Occupational Health and Safety (ANSES), Lyon, France; Epidemiology and Surveillance Support Unit, University of Lyon—French Agency for Food, Environmental and Occupational Health and Safety (ANSES), Lyon, France; UFR des Sciences Pharmaceutiques et Biologique, Université de Montpellier, Montpellier, France; Epidemiology and Surveillance Support Unit, University of Lyon—French Agency for Food, Environmental and Occupational Health and Safety (ANSES), Lyon, France; Epidemiology, Health and Welfare Unit, French Agency for Food, Environmental and Occupational Health and Safety (ANSES), Ploufragan, France; Unité Antibiorésistance et Virulence Bactériennes, ANSES—Université de Lyon, Lyon, France; Epidemiology and Surveillance Support Unit, University of Lyon—French Agency for Food, Environmental and Occupational Health and Safety (ANSES), Lyon, France; Unité Antibiorésistance et Virulence Bactériennes, ANSES—Université de Lyon, Lyon, France; French Agency for Veterinary Medicinal Products, ANSES, Fougères, France; Epidemiology and Surveillance Support Unit, University of Lyon—French Agency for Food, Environmental and Occupational Health and Safety (ANSES), Lyon, France

## Abstract

**Objectives:**

In France, a 10 year national policy to reduce antibiotic use (ABU) in animals led, respectively, to an 88% and 94% decrease in animal exposure to fluoroquinolones and third- and fourth-generation cephalosporins (3GC-4GC) between 2011 and 2021.

**Methods:**

Building on national surveillance data, this study evaluated the impact of this national policy on reducing the occurrence of resistance to fluoroquinolones and 3GC-4GC in clinical *Escherichia coli* from young cattle and dogs and cats in France. The effect of homologous and heterologous use of antibiotics was explored using multivariate regression modelling. In addition, the time lag between implementation of national policies and subsequent reduction in antibiotic resistance (ABR) was estimated using segmented regression analysis.

**Results:**

Statistical analysis of available ABU and ABR surveillance data demonstrated a significant and sustainable impact on the occurrence of resistance to fluoroquinolones in clinical *E. coli* (in cattle and in dogs and cats), and to a lesser extent to 3GC-4GC (in cattle). The effects were fast and observed within 1 (in cattle) or 2 years (in dogs and cats) after the implementation of the 2014 national policy targeting specifically the animals’ exposure to 3GC-4GC and fluoroquinolones.

**Conclusions:**

The French 10 year national policy had a successful impact on the occurrence of resistance to fluoroquinolones and 3GC-4GC in clinical *E. coli*. A potential shift in ABU from the use of fluoroquinolones and 3GC-4GC to other antibiotic classes, such as trimethoprim/sulfonamides (since 2012), and penicillins and tetracyclines (since 2016), was also observed.

## Introduction

Antimicrobial resistance (AMR) represents a significant threat for public health globally, with an estimated burden of 4.95 million deaths associated with bacterial AMR in 2019, including 1.27 million deaths directly attributable to bacterial AMR.^1^ Mitigation efforts require action from all sectors and actors. In the EU, the European One Health Action Plan against AMR stressed the need to promote the appropriate and prudent use of antimicrobials to limit the emergence of AMR in human healthcare and in animal husbandry.^2^ In the animal sector, the 2020 Farm to Fork Strategy associated to the European Green Deal established the objective to reduce overall EU sales of antimicrobials for farmed animals and in aquaculture by 50% by 2030.^3^ Fluoroquinolones and third- and fourth-generation cephalosporins (3GC-4GC) have been classified by the EMA in the ‘restrict’ category of antibiotics that should be considered only when there are no antibiotics in categories of lower importance (‘caution’ and ‘prudence’ categories) that could be clinically effective.^4^ It is recommended that their use be based on the results of antimicrobial susceptibility testing (AST).

In France, successive national action plans, called Ecoantibio, have been implemented to mitigate AMR in the animal sector. Among 40 mitigation measures included in the first Ecoantibio plan (2012–2017), an objective was set to reduce by 25% the overall animal level of exposure to antimicrobials (ALEA) between 2011 and 2016.^5^ Subsequently, the national law on the future of agriculture, food and forestry adopted in 2014 defined an additional objective to reduce animal level of exposure to fluoroquinolones and 3GC by 25% between 2013 and 2016.^6^ In 2016, the decree no. 2016-317 prohibited the preventive use of 3GC-4GC and fluoroquinolones in animals, and made AST mandatory prior to prescription and delivery of 3GC-4GC and fluoroquinolones to treat animal infections.^7^ Reduction objectives were achieved, with a reduction of 37% of the overall level of animal exposure to antimicrobials between 2011 and 2016, and a reduction of 81% and 75% of animal exposure to 3GC-4GC and fluoroquinolones, respectively, between 2013 and 2016.^8^ The Ecoantibio 2 (2017–2021) plan continued to reinforce this trend, with further reduction of the overall level of animal exposure to antimicrobials by 16.5% between 2016 and 2021.^9^

The overall extent of reduction in animal exposure to antibiotics in France, however, varied between animal species, and was less pronounced for cattle and dogs/cats compared with pigs and poultry, for which a concomitant reduction in the occurrence of resistance to antimicrobials had been observed. As an example, Verliat *et al*.^10^ in 2021 reported a statistically significant positive association between the sales of 3GC-4GC and the occurrence of non-susceptibility to 3GC in both clinical and commensal *Escherichia coli* isolates from pigs, following a marked 98% decrease in pig exposure to 3GC-4GC between 2010 and 2017. Similarly, Perrin-Guyomard *et al*.^11^ in 2020 reported a significant decrease in the proportion of *E. coli* clinical isolates resistant to fluoroquinolones in poultry and pigs, following a reduction in fluoroquinolone exposure by 71.5% in poultry and 89.7% in pigs between 2011 and 2018. However, the impact was less clear on fluoroquinolone resistance of *E. coli* and *Campylobacter jejuni* isolated from healthy animals at slaughter. To our knowledge, the statistical associations between antibiotic use (ABU) and antibiotic resistance (ABR) in the other animal species being monitored in France have not been explored so far. In addition, a potential shift from the use of 3GC-4GC and fluoroquinolones to other antibiotic classes also deserves further attention.

Hence, the objective of this study was to evaluate the impact of a 10 year ABU reduction national policy on the occurrence in France of ABR to fluoroquinolones and 3GC-4GC in clinical *E. coli* from young cattle (<24 months) and from dogs and cats. More specifically, we aimed to: (i) assess the statistical significance of ABU and ABR trends over the time period of interest (2012–2021); (ii) evaluate the statistical associations between ABU and ABR, including potential co-selection effects from use of various antibiotic classes; (iii) describe the time lags between the implementation of new legislation and the subsequent reduction in ABR; and (iv) explore a potential shift from the use of 3GC-4GC and fluoroquinolones to other antibiotic classes.

## Material and methods

### Data sources

ABU data originated from the national sales monitoring of veterinary medicinal products containing antimicrobials in France.^9^ Data were expressed in terms of ALEA (Animal Level of Exposure to Antimicrobials), which corresponds to the ratio between the estimated body weight treated and the biomass of the animal population potentially using antibiotics. Sales data are reported annually by the marketing authorization holders, who also provide an estimate of the antibiotic sales breakdown per animal species. However, data for dogs and cats were grouped together, since marketing authorization holders were unable to distinguish antibiotic sales between the two species. Similarly, ABU data in cattle included sales data in all age groups in the absence of ABU data specific to young cattle, and to capture a potential impact of ABU in cows on ABR in their offspring.

ABR data originated from the RESAPATH French network for surveillance of ABR in bacteria from diseased animals,^12^ which is a large surveillance network centralizing data on AST of clinical isolates from both livestock and companion animals, generated via routine diagnostic activities of a growing number of public and private veterinary laboratories participating on a voluntary basis (64 laboratories in 2012, 101 laboratories in 2021). All laboratories follow AST disc diffusion guidelines and interpretation criteria from the antibiogram committee of the French Society of Microbiology,^13^ and participate in annual inter-laboratory proficiency testing organized by the French Agency for Food, Environmental and Occupational Health Safety (ANSES). Resistance (including both intermediate and resistant profiles) to at least one of the antibiotic agents tested was considered as resistance to the antibiotic class (Table S1, available as Supplementary data at *JAC-AMR* Online). In order to obtain a baseline situation prior to the ABU reduction that started in the early 2010s, historical ABR data were included with the condition to have at least 30 available *E. coli* isolates per year and animal species. Hence, the time period retained for this study was 2002–2021 for young cattle (<24 months), and 2007–2021 for dogs and cats. The annual number of isolates included in the study are shown in Figures S1 and S2. Of note, data originating from the national active monitoring of ABR in cattle at slaughter and food^14^ were excluded from this study, considering the limited number of timepoints available for cattle data over the time period of interest (namely 2015, 2017, 2019, 2021).

### Analysis of ABU and ABR trends

**Figure 1.**
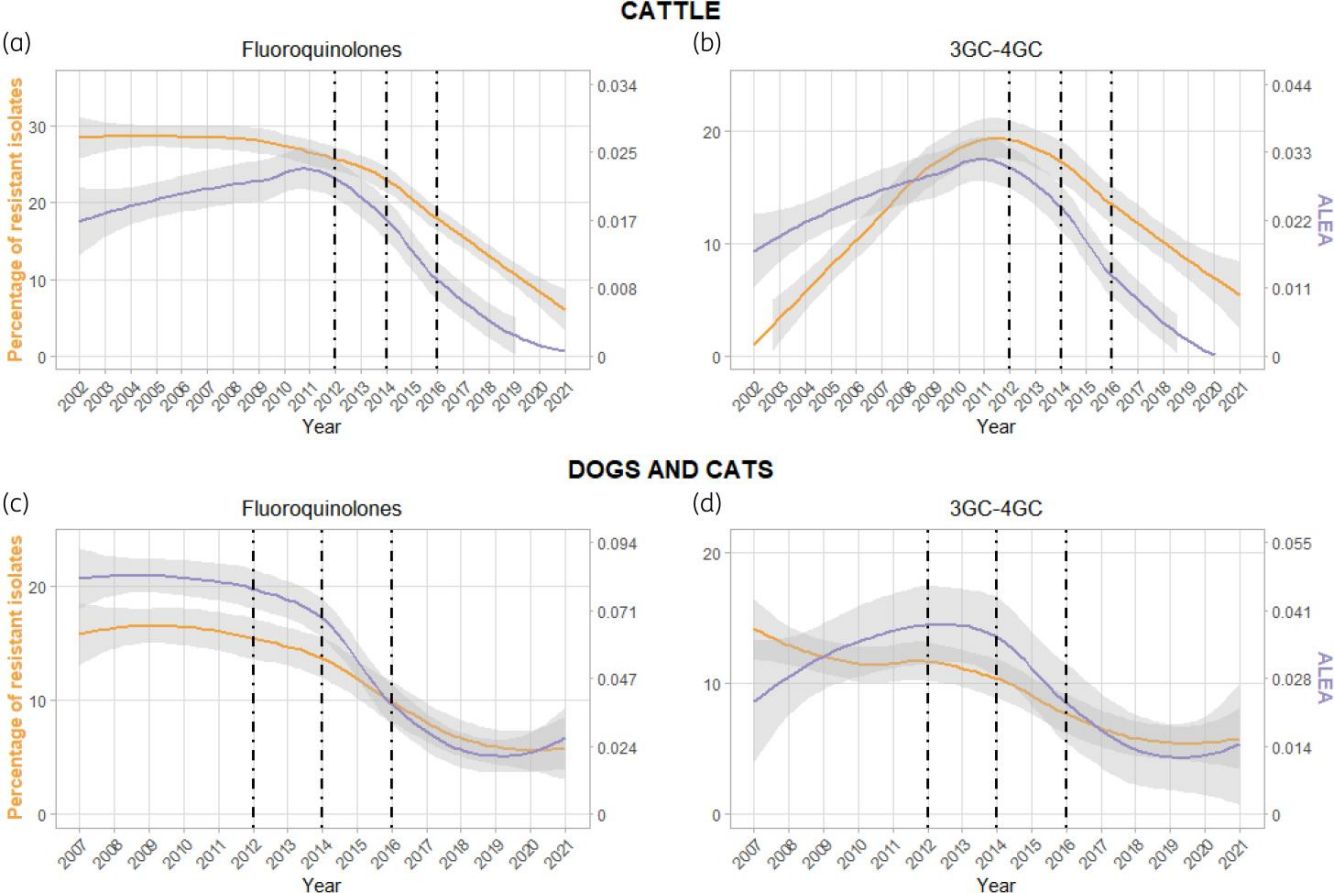
Trends of antibiotic resistance (yellow line) and antibiotic use (purple line) over time. (a) Trends of resistance to fluoroquinolones and consumption of fluoroquinolones in cattle. (b) Trends of resistance to third-generation cephalosporins (3GC) and consumption of third- and fourth-generation cephalosporins (3GC-4GC) in cattle. (c) Trends of resistance to fluoroquinolones and consumption of fluoroquinolones in dogs and cats. (d) Trends of resistance to 3GC and consumption of 3GC-4GC in dogs and cats. Grey zone around the curve represents the 95% CI. Dashed black vertical lines represent the years of implementation of national policy interventions (namely 2012 for Ecoantibio1, 2014 for the national law on the future of agriculture, food and forestry, and 2016 for the decree no. 2016-317 on 3GC-4GC and fluoroquinolones). Of note, ABU data were smoothed over 2014–2015 to correct for an artefact due to a regulatory change that induced stockpiling in 2014 of antibiotics used only in 2015. ALEA, animal level of exposure to antimicrobials.

For each animal species and antibiotic of interest, ABR trends are displayed against ABU trends on the same graph (Figure 1). The statistical significance of ABU trends over time was assessed using linear regression modelling. The best-fit model was selected between a simple linear regression model (Equation 1) and a second-order polynomial regression model (Equation 2), based on the highest adjusted *R*^2^.


(1) ABU=αyear+ε

(2) $$\mathrm{ABU}=\alpha_{1}\;\mathrm{year}+\alpha_{2}\;{\mathrm{year}}^{2}+\varepsilon$$

where *ε* represent the residuals of the model.

Model validation was performed using Shapiro–Wilk testing to check for normal distribution of the residuals, Breusch–Pagan testing for heteroscedasticity, and Durbin–Watson testing for autocorrelation in the residuals, using a *P* value ≤0.05 as a threshold.

A similar approach was used to assess the significance of ABR trends over time, this time using a negative binomial generalized linear model with a log link to control for over-dispersion in the residuals (Equations 3 and 4). An offset was introduced to scale the model and take into account the time-varying number of isolates.


(3) Ln(R)=αyear+offset(log(R+S))+ε

(4) $$\mathrm{Ln}(R)=\alpha_{1}\;\mathrm{year}+\alpha_{2}\;{\mathrm{year}}^{2}+\mathrm{offset}(\log(R+S))+\varepsilon$$

where R is the number of resistant isolates, S the number of susceptible isolates and *ε* the residuals of the model. Hence, R + S represents the number of isolates tested against the antibiotic of interest.

### Association between ABR and ABU

Statistical associations between ABR and ABU were explored using multivariate negative binomial modelling with a log link for every combination of antibiotic class or agent with its corresponding resistance, adjusting for the use of other antibiotics, and for year. The best-fit model was selected between a simple linear multivariate regression model (Equation 5) and a second-order polynomial multivariate regression model (Equation 6), based on smallest Akaike information criterion (AIC).


(5) $$\mathrm{Ln}(R)=\beta_{0}+\beta_{1}\;{\mathrm{ABU}}_{1}+\beta_{2}\;{\mathrm{ABU}}_{2}+\ldots +\beta_{k}\;{\mathrm{ABU}}_{k}+\;\alpha \;\mathrm{year}+\mathrm{offset}(\log(R+S))+\varepsilon$$

(6) $$\mathrm{Ln}(R)=\beta_{0}+\beta_{1}\;{\mathrm{ABU}}_{1}+\beta_{2}\;{\mathrm{ABU}}_{2}+\ldots +\beta_{k}\;{\mathrm{ABU}}_{k}+\alpha_{1}\;\mathrm{year}+\alpha_{2}\;{\mathrm{year}}^{2}+\mathrm{offset}(\log(R+S))+\varepsilon$$

where R is the number of resistant isolates (including both resistant and intermediate profiles), S the number of susceptible isolates, ABU_k_ the level of ABU for an antibiotic class or agent k (in the same year as R and S), and *ε* the residuals of the model. Practically, *β_k_* estimates the increase in Ln(R) observed for every increase by 1 unit of ALEA×1000.

ABU_k_ corresponding to the ABR being studied (i.e. homologous antibiotic use) was systematically included to facilitate biological interpretation of the results. ABU_k_ other than the one being studied (i.e. heterologous use from any other antibiotic class) were also included to account for potential co-selection effects. ABU_k_ primary selection was based on statistical significance of the univariate model at a level of 0.1. Multi-collinearity between variables was tested with the variance inflator factor (VIF). Although there is no agreed benchmark for the VIF we determined that a VIF >5 would be cause for concern. A *P* value of ≤0.05 was considered as statistically significant in the final multivariate model. Validation of the multivariate model was performed using the same approach as for the trends analysis.

### Identification of breaking timepoints

**Figure 2.**
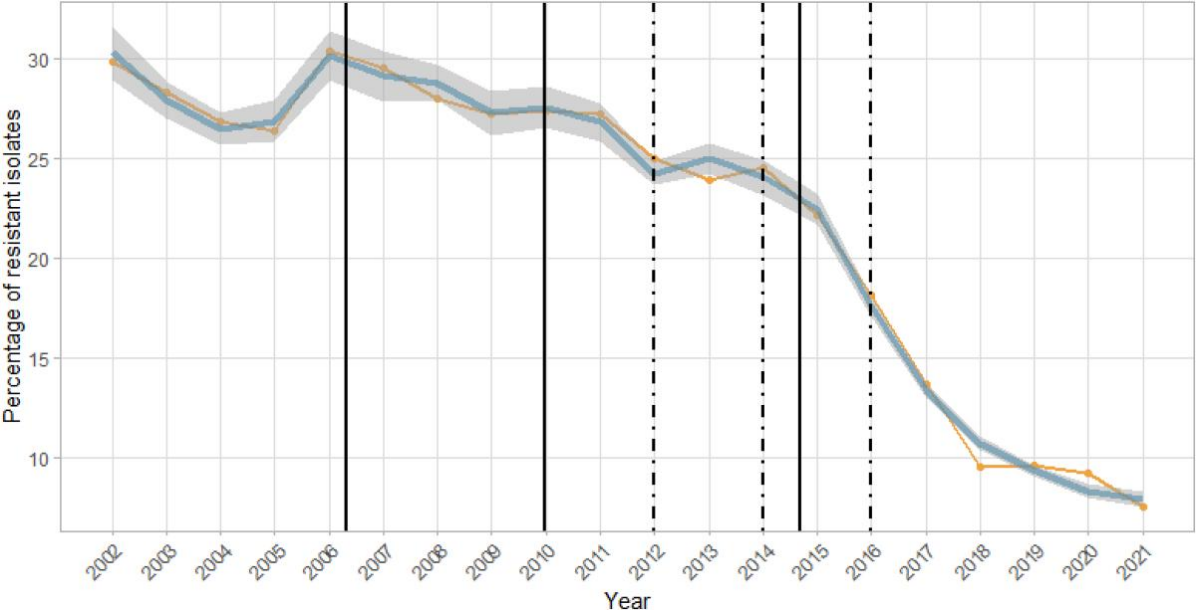
Segmented regression of *E. coli* clinical isolates resistant to fluoroquinolones in young cattle over 2002–2021. The yellow curve represents the observed resistance percentage, and the blue curve represents the resistance percentage as predicted by the multivariate regression model together with its 95% CI (grey area around the curve). The dashed black vertical lines represent the years of implementation of national policy interventions (namely 2012 for Ecoantibio 1, 2014 for the national law on the future of agriculture, food and forestry, and 2016 for the decree no. 2016-317 on 3GC-4GC and fluoroquinolones). The solid black vertical lines represent the breaking timepoints estimated by the segmented regression model. CIs around the breaking timepoints are presented in Tables S2 to S5.

Building on the multivariate negative binomial models described above, segmented regression analysis was used to identify breaking timepoints at which trends in the ABR model (i.e. *β_k_* in Equations 5 and 6) significantly changed.^15^ The expected number of breaking timepoints was fixed by default to three based on the number of national interventions implemented over the time period of interest. However, only two breaking timepoints were used in dogs and cats to adjust for convergence issues. The years of implementation of these interventions were 2012 for the national action plan Ecoantibio1, 2014 for the national law on the future of agriculture, food and forestry, and 2016 for the decree no. 2016-317 on 3GC-4GC and fluoroquinolones. These years were compared against the estimated breaking timepoints, in order to assess the time lag between implementation and observed impact (or breach) on ABR trends (Figure 2).

All data analyses were performed in R studio version 4.2.3.^16^ Segmented regression analysis was conducted using the {segmented} package version 2.1-4.

## Results

### Descriptive trends of ABU and ABR

Trends of ABU and ABR in *E. coli* for fluoroquinolones and 3GC-4GC in cattle, dogs and cats are displayed in Figure 1. Between 2012 and 2021, the use of fluoroquinolones was cut by 89.9% in cattle and by 66.4% in dogs and cats, and the use of 3GC-4GC was cut by 95.1% in cattle and by 55.9% in dogs and cats. Associated ABU and ABR trends proved to be highly significant (Tables 1 and 2). Concomitantly, a significant increase in the use of trimethoprim/sulfonamides was observed between 2012 and 2021 in both cattle and dogs and cats. Conversely, a significant decrease was observed in the use of polymyxins in both cattle and dogs and cats, and in the use of aminoglycosides and penicillins in dogs and cats (Table 1 and Figures S3 to S13).

**Table 1.** Significance of ABU trends over the period 2012–2021 in cattle, and in dogs and cats

Animal species	Antibiotic class or agent	Statistical model^a^	Regression coefficient^a^	Trend estimate	*P* value^c^
Cattle	Fluoroquinolones	M2	*α_1_* *α_2_*	−1656 0.41	**0.021** **0.021**
	3GC-4GC^b^	M2	*α_1_* *α_2_*	−2363 0.58	**0.013** **0.013**
	Aminoglycosides	M2	*α_1_* *α_2_*	−2640 −0.6546	Non-significant
	Penicillins	M1	*α_1_*	1.1558	Non-significant
	Phenicols	M1	*α_1_*	0.2362	Non-significant
	Polymyxins	M1	*α_1_*	−1.1194	**0.044**
	Tetracyclines	M2	*α_1_* *α_2_*	−66.83 0.01669	Non-significant
	Trimethoprim/sulfonamides	M1	*α_1_*	1.1564	**<0.001**
Dogs and cats	Fluoroquinolones	M1	*α*	−7.17	**<0.001**
	3GC-4GC^b^	M1	*α*	−3.26	**0.009**
	Aminoglycosides	M2	*α_1_* *α_2_*	−9702 2.404	**0.030** **0.030**
	Penicillins	M2	*α_1_* *α_2_*	−14 920 3.7	**0.033** **0.033**
	Polymyxins	M1	*α_1_*	−0.44372	**<0.001**
	Tetracyclines	M2	*α_1_* *α_2_*	−1440 0.3576	Non-significant
	Trimethoprim/sulfonamides	M2	*α_1_* *α_2_*	2721 −0.6741	**0.014** **0.014**

^a^M1: linear regression model (see Equation 1); M2: second-order polynomial regression model (see Equation 2).

^b^Third- and fourth-generation cephalosporins.

^c^Bold type denotes statistical significance.

**Table 2.** Significance of *E.coli* ABR trends over the period 2012–2021 in young cattle, and in dogs and cats

Animal species	Antibiotic class or agent to which *E. coli* is resistant	Statistical model^a^	Regression coefficient^a^	Trend estimate	*P* value^c^
Cattle	Fluoroquinolones	M3	*α*	−0.15	**<0.001**
	3GC-4GC^b^	M3	*α*	−0.14	**<0.001**
Dogs and cats	Fluoroquinolones	M3	*α*	−0.13	**<0.001**
	3GC-4GC^b^	M4	*α_1_* *α_2_*	−44.64 0.01	**0.033** **0.034**

^a^M3: negative binomial model (see Equation 3); M4: second-order polynomial negative binomial model (see Equation 4).

^b^Third- and fourth-generation cephalosporins.

^c^Bold type denotes statistical significance.

### Multivariate regression modelling

The results of multivariate regression modelling are presented in Tables 3 and 4 for cattle, and Tables 5 and 6 for dogs and cats. In both cattle and dogs and cats, *E. coli* resistance to fluoroquinolones was positively associated with fluoroquinolone use over the time period of interest, and after controlling for the effect of time (Tables 3 and 5). In cattle, sales of aminoglycosides were negatively associated with *E. coli* resistance to fluoroquinolones.

**Table 3.** Results of the multivariate negative binomial model for *E. coli* resistance to fluoroquinolones in young cattle over the period 2002–2021^a^

	Coefficient	Standard error	*P* value^b^
Intercept	−2.27 × 10^4^	3.79 × 10^3^	**<0.001**
ABU fluoroquinolones	1.05 × 10^−2^	5.25 × 10^−3^	**0.047**
ABU aminoglycosides	−1.16 × 10^−2^	5.08 × 10^−3^	**0.023**
ABU tetracyclines	−1.25 × 10^−3^	1.52 × 10^−3^	0.413
ABU trimethoprim/sulfonamides	2.30 × 10^−2^	2.01 × 10^−2^	0.254
year	22.60	3.78	**<0.001**
year^2^	−5.64 ×10^−3^	9.40 ×10^−4^	**<0.001**

^a^A second-order polynomial multivariate regression model was used, as described in Equation 6.

^b^Bold type denotes statistical significance.

**Table 4.** Results of the multivariate negative binomial model for *E. coli* resistance to third-generation cephalosporins in young cattle over the period 2002–2021^a^

	Coefficient	Standard error	*P* value^b^
Intercept	−5.06 ×10^4^	9.59 ×10^3^	**<0.001**
ABU 3GC-4GC^c^	2.17 ×10^−2^	7.64 ×10^−3^	**0.005**
ABU aminopenicillins	−4.35 ×10^−3^	2.19 ×10^−2^	0.843
year	50.21	9.54	**<0.001**
year^2^	−1.25 ×10^−2^	2.37 ×10^−3^	**<0.001**

^a^A second-order polynomial multivariate regression model was used, as described in Equation 6.

^b^Bold type denotes statistical significance.

^c^Sales of third- and fourth-generation cephalosporins.

**Table 5.** Results of the multivariate negative binomial model for *E. coli* resistance to fluoroquinolones in dogs and cats over the period 2007–2021^a^

	Coefficient	Standard error	*P* value^b^
Intercept	61.22	48.30	0.205
ABU fluoroquinolones	1.11 × 10^−2^	3.65 × 10^−3^	**0**.**002**
ABU tetracyclines	−2.77 × 10^−3^	7.20 × 10^−3^	0.700
ABU trimethoprim/sulfonamides	7.31 × 10^−4^	7.10 × 10^−3^	0.918
year	−3.13 × 10^−2^	2.40 × 10^−2^	0.186

^a^A simple linear multivariate regression model was used, as described in Equation 5.

^b^Bold type denotes statistical significance.

**Table 6.** Results of the multivariate negative binomial model for *E. coli* resistance to third-generation cephalosporins in dogs and cats over the period 2007–2021^a^

	Coefficient	Standard error	*P* value^b^
Intercept	116.80	31.14	**<0.001**
ABU 3GC-4GC^c^	7.08 × 10^−3^	4.81 × 10^−3^	0.141
year	−5.923 × 10^−2^	1.54 × 10^−2^	**<0.001**

^a^A simple linear multivariate regression model was used, as described in Equation 5.

^b^Bold type denotes statistical significance.

^c^Sales of third- and fourth-generation cephalosporins.

Similarly, *E. coli* resistance to 3GC was positively associated with 3GC-4GC sales in cattle (Table 4), but the association was not statistically significant in dogs and cats (Table 6).

### Segmented regression

Results of the segmented regression are displayed in Figure 2 for *E. coli* resistant to fluoroquinolones in cattle. Results of the other combinations are shown in Figure S14. In general, it was difficult to clearly link the estimated breaking timepoints to the years of implementation of national policy interventions. An exception was the 2014 national law on the future of agriculture, food and forestry, for which a breaking timepoint in *E. coli* resistance to fluoroquinolones was observed within 1 year in cattle (Figure 2), and within 2 years in dogs and cats (Figure S14c). Similarly, a breaking timepoint in *E. coli* resistance to 3GC-4GC was observed within 1 year in cattle (Figure S14b). However, in several instances, such as *E. coli* resistance to fluoroquinolones in cattle (Figure 2), several of the estimated breaking timepoints preceded the national policy interventions.

## Discussion

### Main results

This study evaluated the impact of a 10 year reduction effort in critically important ABU on the occurrence of ABR in clinical *E. coli* from young cattle and dogs and cats in France, following the implementation of successive national action plans and policy measures since the early 2010s. Overall, our study demonstrated that both ABU and ABR of fluoroquinolones and 3GC-4GC significantly decreased after interventions. No concomitant increase in the use of other antibiotic classes was observed, with the exception of trimethoprim/sulfonamides, for which ABU significantly increased over 2012–2021 in both cattle and dogs and cats, although it reduced again in dogs and cats from 2018 onwards (Figure S13). This could potentially be due to a shift in ABU from fluoroquinolones and cephalosporins to trimethoprim/sulfonamides. Notably, no increase was observed in clinical *E. coli* resistance to trimethoprim/sulfonamides in cattle and dogs and cats over 2012–2021.^12^ Although not significant over 2012–2021, an increase in the use of penicillins in cattle and dogs and cats (Figures S4 and S10) and tetracyclines in dogs and cats (Figure S12) was also observed after 2016, and could relate to a further switch from the use of fluoroquinolones and last-generation cephalosporins to other antibiotic classes, following the implementation of the decree no. 2016-317 on 3GC-4GC and fluoroquinolones.

The strength of the associations between ABU and ABR differed depending on animal species and antibiotic classes of interest. Significant positive association was observed for fluoroquinolones in both cattle and dogs and cats, in line with previous publications in pigs^11^ and multiple livestock species.^17,18^ Interestingly, the European Food Safety Authority also reported a decrease in fluoroquinolone resistance of commensal *E. coli* isolated from young cattle at slaughter in France over 2013–2021.^14^ This apparent reversibility of fluoroquinolone resistance in clinical *E. coli* following a marked decrease in fluoroquinolone use (i.e. homologous use) could relate to various aspects such as the mechanism of fluoroquinolone resistance acquisition (primarily via target-site mutation)^19,20^ and the associated fitness cost, reported to be high for fluoroquinolone resistance in *E. coli*.^21^ Selection for 3GC-4GC–resistant *E. coli* via homologous use of 3CG-4GC was also supported in cattle, but the relationship was not significant in dogs/cats, potentially because the reduction in 3CG-4GC use was not as great for dogs/cats (55.9%) compared with cattle (95.1%). Existing literature also shows inconsistent effects, e.g. with a positive significant association in pigs^10^ and a non-significant association in multiple livestock species.^18^

Compared with previous similar literature such as Dorado-García *et al*.,^17^ a strength of our study was the ability to explore which antibiotic classes could be involved in co-selection of resistance, rather than the overall effect of total ABU. However, we found limited evidence for co-selection of resistance in our models. Interestingly, fluoroquinolone-resistant *E. coli* in cattle was negatively associated with aminoglycoside use. Further exploration of RESAPATH data showed that aminoglycoside-resistant *E. coli* in cattle tended to be more frequently susceptible to fluoroquinolones (data not shown). However, this result might differ depending on aminoglycoside molecules, this class being heterogeneous in terms of resistance mechanisms.

The segmented analysis showed that the frequency of fluoroquinolone-resistant *E. coli* quickly reduced (within 1 year in cattle, within 2 years in dogs and cats) after the implementation of the 2014 national law on the future of agriculture, food and forestry. A similar result was observed for 3GC-4GC–resistant *E. coli* in cattle. However, the other breaking timepoints could not be clearly linked to a national policy. Hence, not all three national policies have been followed by a breach in fluoroquinolone and 3GC-4GC resistance trends. One reason could be that the 2014 national law on the future of agriculture, food and forestry had a clear focus on restricting the use of fluoroquinolones and 3GC-4GC in animals.^6^ Conversely, Ecoantibio1 had a broader scope and aimed to reduce overall veterinary antibiotic use.

### Limitations

The main limitation for this study was inherent to the data sources being used. ABR data originated from the RESAPATH passive surveillance network, for which coverage and data volume have been increasing over time (Figures S1 and S2). This may have somewhat impaired the comparability of resistance proportions over time, and the impact assessment in dogs and cats, where the study period was reduced compared with cattle. However, the standards for AST testing and interpretation have been consistent throughout the study period. Another limitation was the use of phenotypic ABR resistance profiles, potentially combining heterogeneous molecular mechanisms of resistance, with various levels of association to ABU. Identifying clonal or molecular evolutions driving ABR trends would be of high interest but was beyond the scope of this study.

ABU data were limited by our inability to distinguish young from adult cattle, and dogs from cats. In addition, ABU data were only available at the antibiotic class level, hence limiting our ability to explore ABU-ABR associations with more scrutiny, at the antibiotic molecule level. ABU data relied on antibiotic sales data, which are only a proxy for actual use, with various sources of uncertainty (e.g. estimates from market authorization holders on the sales breakdown per animal species). As an example, antibiotic sales artificially increased in 2014 due to stockpiling of antibiotic products following the introduction of the national law on the future of agriculture, food and forestry; some of these were used in 2015.^9^ Smoothing was used during data analysis to address this artefact. In addition, a lag of 1 year between ABU and ABR was tested but had no impact on the model results (data not shown).

Another limitation of this study related to our ability to link breaches in *E. coli* resistance trends and national policy implementation. The R function used to estimate the breaking timepoints in ABR trends requires the number of breaking timepoints to be informed. We used three by default to align with the number of national policies (two in the dogs and cats models because of convergence issues). However, it seems several breaking timepoints preceded (by several years) national policies and hence were unrelated to them. In addition, the estimation of the time lag between national policy implementation and reduction in resistance levels was likely biased by other events, e.g. the implementation of local voluntary ABU reduction measures, or the progressive implementation of national policies prior to their official date of entry into force.

### Perspectives

Further improvements include the use of more precise and frequent ABU data, available at animal species and at sub-national level. These should become available in the near future, via the so called CalypsoVet national surveillance system,^22^ which has been developed from 2023 onwards to collect prescriptions and deliveries data from veterinarians and pharmacists, in accordance with the new EU regulation 2019/6 on veterinary medicinal products.^23^ These data could also enable the extension of the approach to other animal species such as equids, where ABU sales data currently lack precision.

To improve our understanding of the evolution and trends of antibiotic-resistant *E. coli* over time, another perspective would be to perform WGS on selected isolates of interest. Priority could be given to *E. coli* resistant to 3GC, for which clonal populations and resistant mechanisms are highly diverse and rather complex because of the role of mobile genetic elements. Hence, similar statistical models could be developed using specific *E. coli* sequence types or plasmids carrying ABR genes, rather than overall 3GC-resistant *E. coli*.

Beyond the impact of ABU reduction on ABR in animals, an extension of this study could be to explore the impact on resistant *E. coli* in human populations. A systematic review and meta-analysis conducted by Tang *et al*.^24^ in 2017 reported a 24% lower pooled prevalence of ABR in human studies with interventions on restricting the use of antibiotics in food-producing animals compared with control groups, with a stronger association seen for humans with direct contact with food-producing animals, and a much less clear impact for the general human population. However, this impact is likely to depend on multiple factors, such as the geographical location and the drug-bug combinations of interest, as described by the European integrated analysis of ABU and ABR in bacteria from humans and food-producing animals.^18^

### Conclusion

This study evaluated the impact of a 10 year national policy to reduce critically important ABU on the occurrence of ABR in clinical *E. coli* from cattle and dogs and cats in France. Statistical analysis of available ABU and ABR surveillance data demonstrated a massive, rapid and sustainable impact on the occurrence of resistance to fluoroquinolones in clinical *E. coli* (in cattle, dogs and cats), and to a lesser extent to 3GC-4GC (in cattle). A potential shift in ABU from fluoroquinolones and 3GC-4GC to other antibiotic classes, such as trimethoprim/sulfonamides (since 2012), and penicillins and tetracyclines (since 2016), was also observed.

## Supplementary Material

dlaf207_Supplementary_Data

## Supplementary data

Figures S1 to S14 and Tables S1 to S5 are available as Supplementary data at *JAC-AMR* Online.
