# Supplementary material for: Evaluating the impact of a 10 year reduction in critically important antibiotic use on the occurrence of antibiotic resistance in E. coli from cattle, dogs and cats in France
Source: JAC Antimicrob Resist. 2025 Nov 18;7(6):dlaf207. doi: 10.1093/jacamr/dlaf207 (PMC12624393; doi:10.1093/jacamr/dlaf207)
Supplement: dlaf207_Supplementary_Data [file dlaf207_supplementary_data.docx]

**Supplementary material**

**
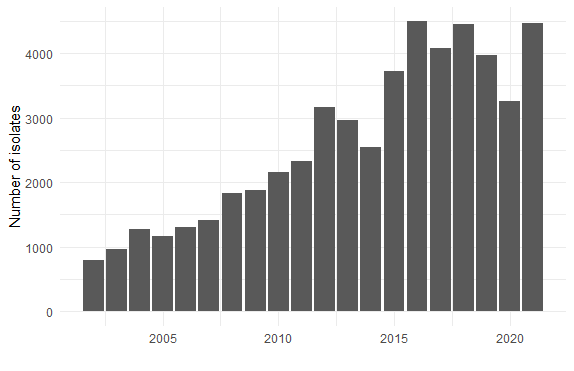
**

**Figure S1.** Distribution of the number of *E. coli* isolates tested for susceptibility to antimicrobials over 2002-2021 in young cattle and included in the study (Source: RESAPATH)

**
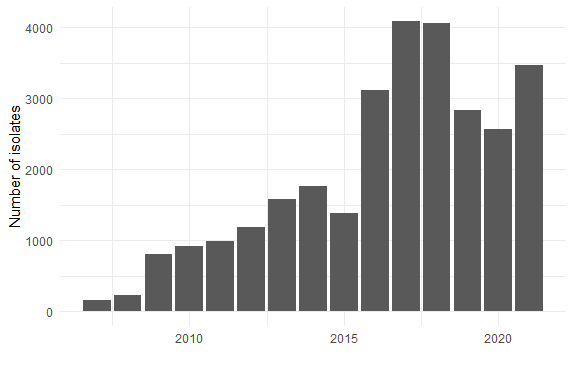
**

**Figure S2.** Distribution of the number of *E. coli* isolates tested for susceptibility to antimicrobials over 2007-2021 in dogs and cats and included in the study (Source: RESAPATH)


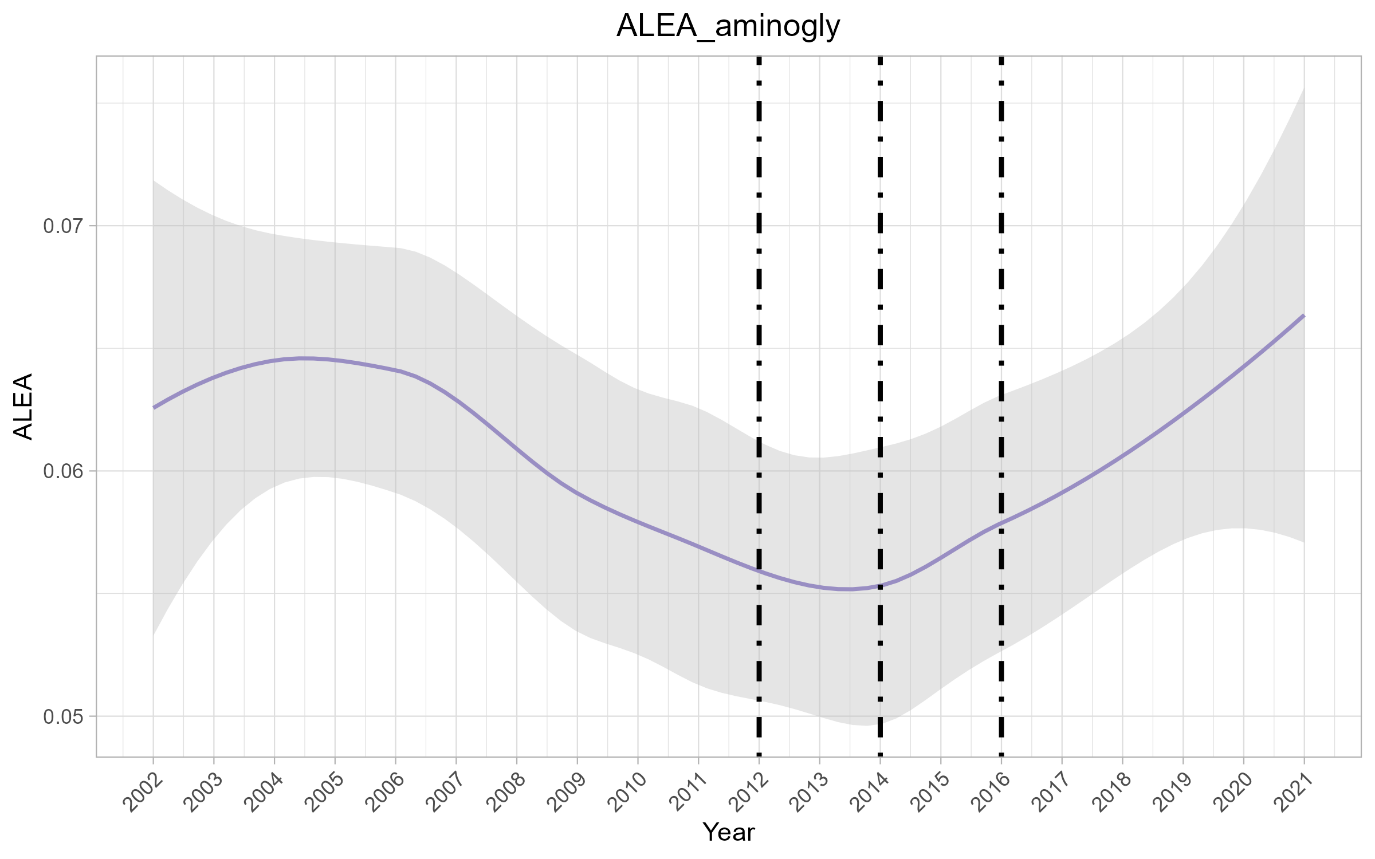


**Figure S3.** Trends of aminoglycosides use in cattle over 2002-2021, expressed as ALEA. Source: Anses 2022^9^

ALEA; animal level of exposure to antimicrobials. Grey zone around the curve represents the 95% confidence interval. Dashed black vertical lines represent the years of implementation of national policy interventions (namely 2012 for Ecoantibio1, 2014 for the national law on the future of agriculture, food and forestry, and 2016 for the decree n° 2016-317 on 3GC-4GC and fluoroquinolones)


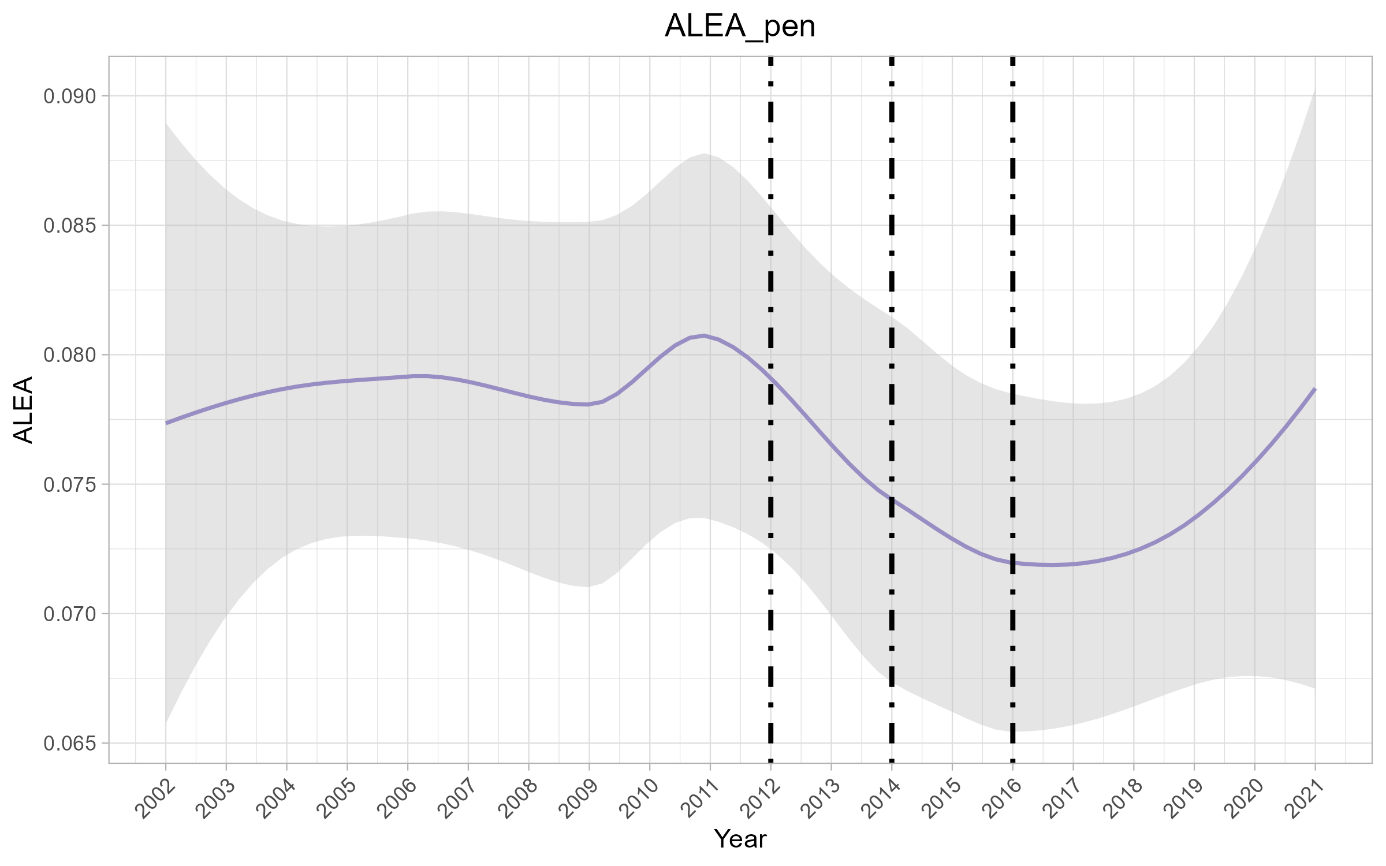


**Figure S4.** Trends of penicillins use in cattle over 2002-2021, expressed as ALEA. Source: Anses 2022^9^

ALEA; animal level of exposure to antimicrobials. Grey zone around the curve represents the 95% confidence interval. Dashed black vertical lines represent the years of implementation of national policy interventions (namely 2012 for Ecoantibio1, 2014 for the national law on the future of agriculture, food and forestry, and 2016 for the decree n° 2016-317 on 3GC-4GC and fluoroquinolones)


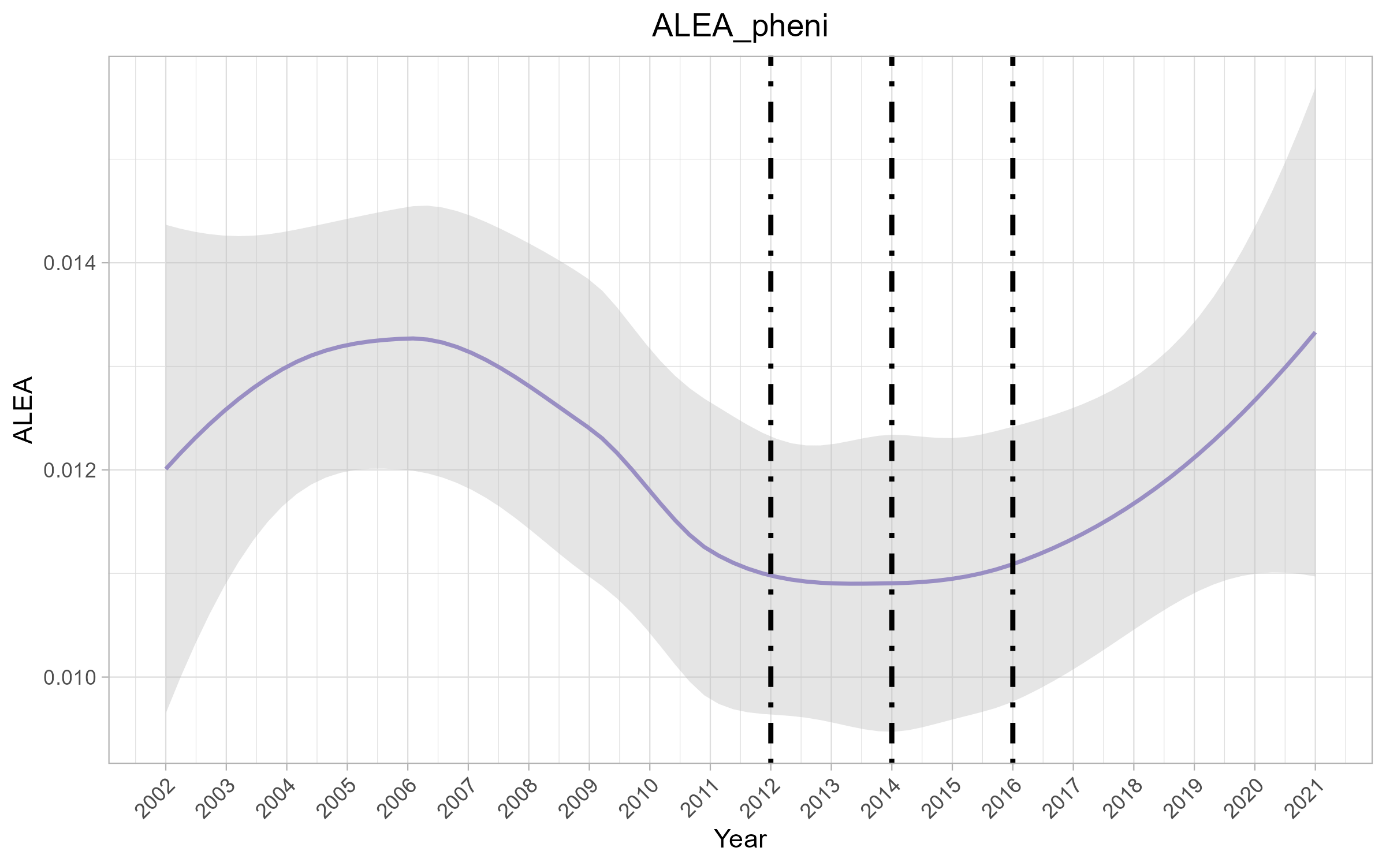


**Figure S5.** Trends of phenicols use in cattle over 2002-2021, expressed as ALEA. Source: Anses 2022^9^

ALEA; animal level of exposure to antimicrobials. Grey zone around the curve represents the 95% confidence interval. Dashed black vertical lines represent the years of implementation of national policy interventions (namely 2012 for Ecoantibio1, 2014 for the national law on the future of agriculture, food and forestry, and 2016 for the decree n° 2016-317 on 3GC-4GC and fluoroquinolones)


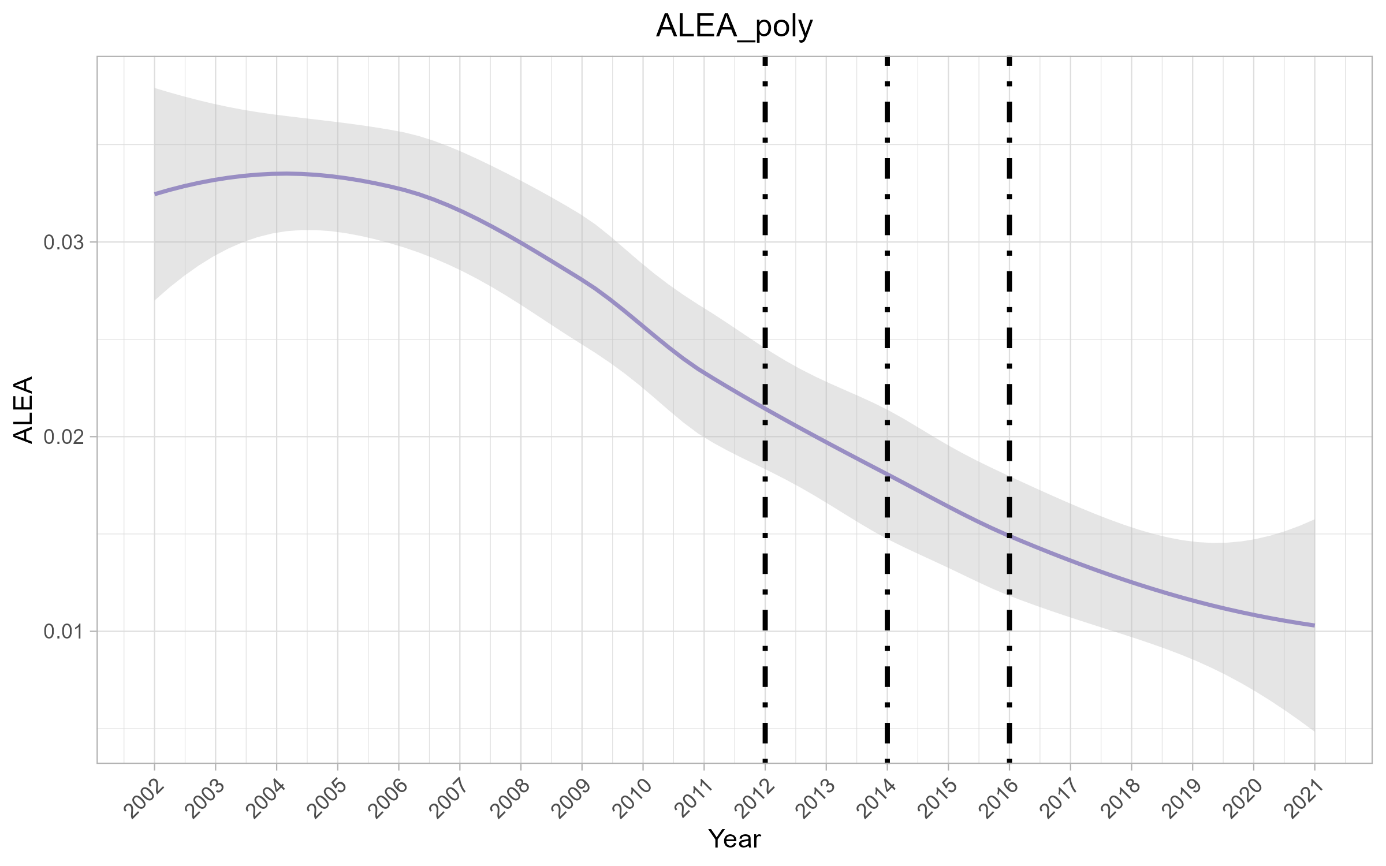


**Figure S6.** Trends of polymyxins use in cattle over 2002-2021, expressed as ALEA. Source: Anses 2022^9^

ALEA; animal level of exposure to antimicrobials. Grey zone around the curve represents the 95% confidence interval. Dashed black vertical lines represent the years of implementation of national policy interventions (namely 2012 for Ecoantibio1, 2014 for the national law on the future of agriculture, food and forestry, and 2016 for the decree n° 2016-317 on 3GC-4GC and fluoroquinolones)


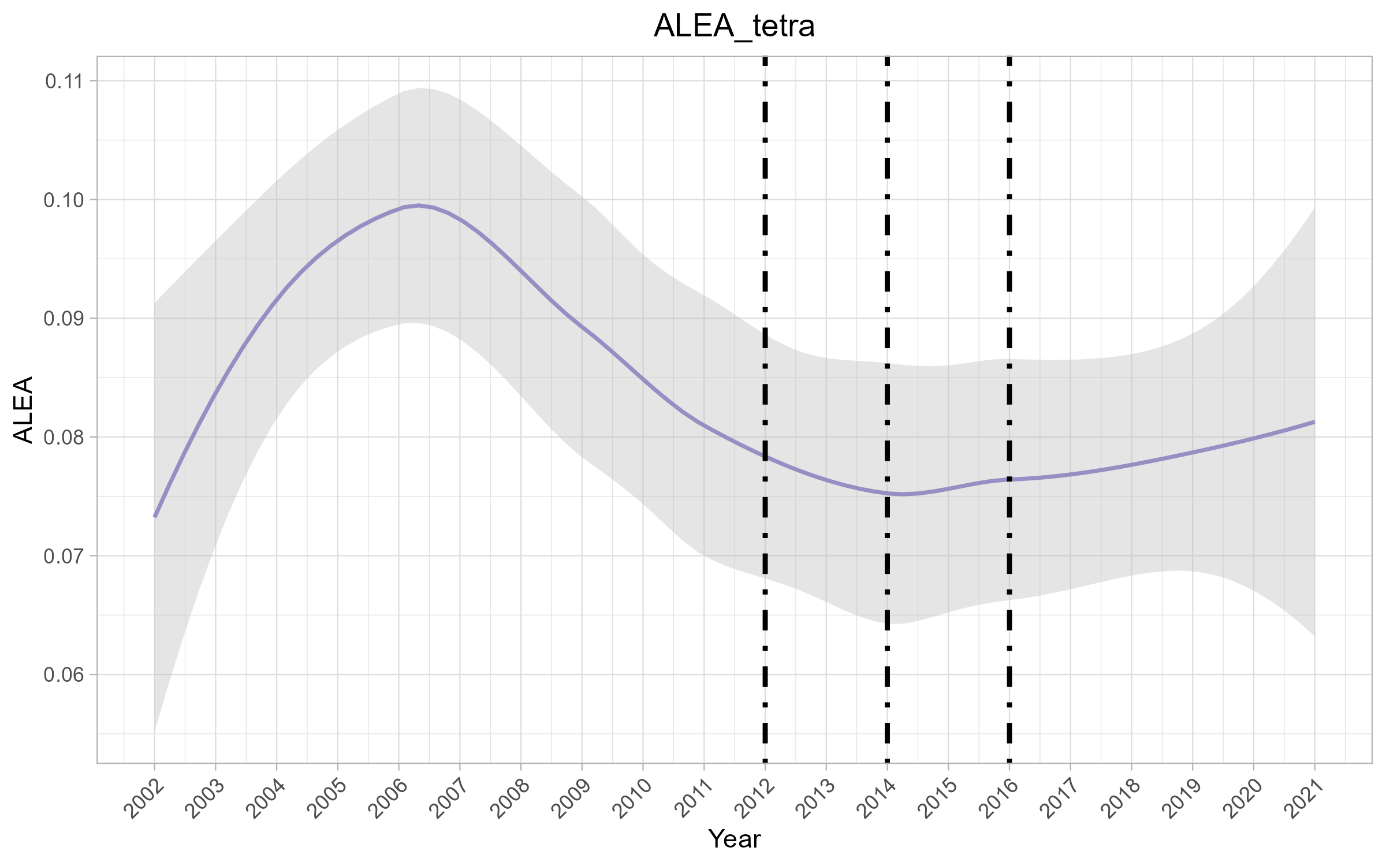


**Figure S7.** Trends of tetracyclines use in cattle over 2002-2021, expressed as ALEA. Source: Anses 2022^9^

ALEA; animal level of exposure to antimicrobials. Grey zone around the curve represents the 95% confidence interval. Dashed black vertical lines represent the years of implementation of national policy interventions (namely 2012 for Ecoantibio1, 2014 for the national law on the future of agriculture, food and forestry, and 2016 for the decree n° 2016-317 on 3GC-4GC and fluoroquinolones)


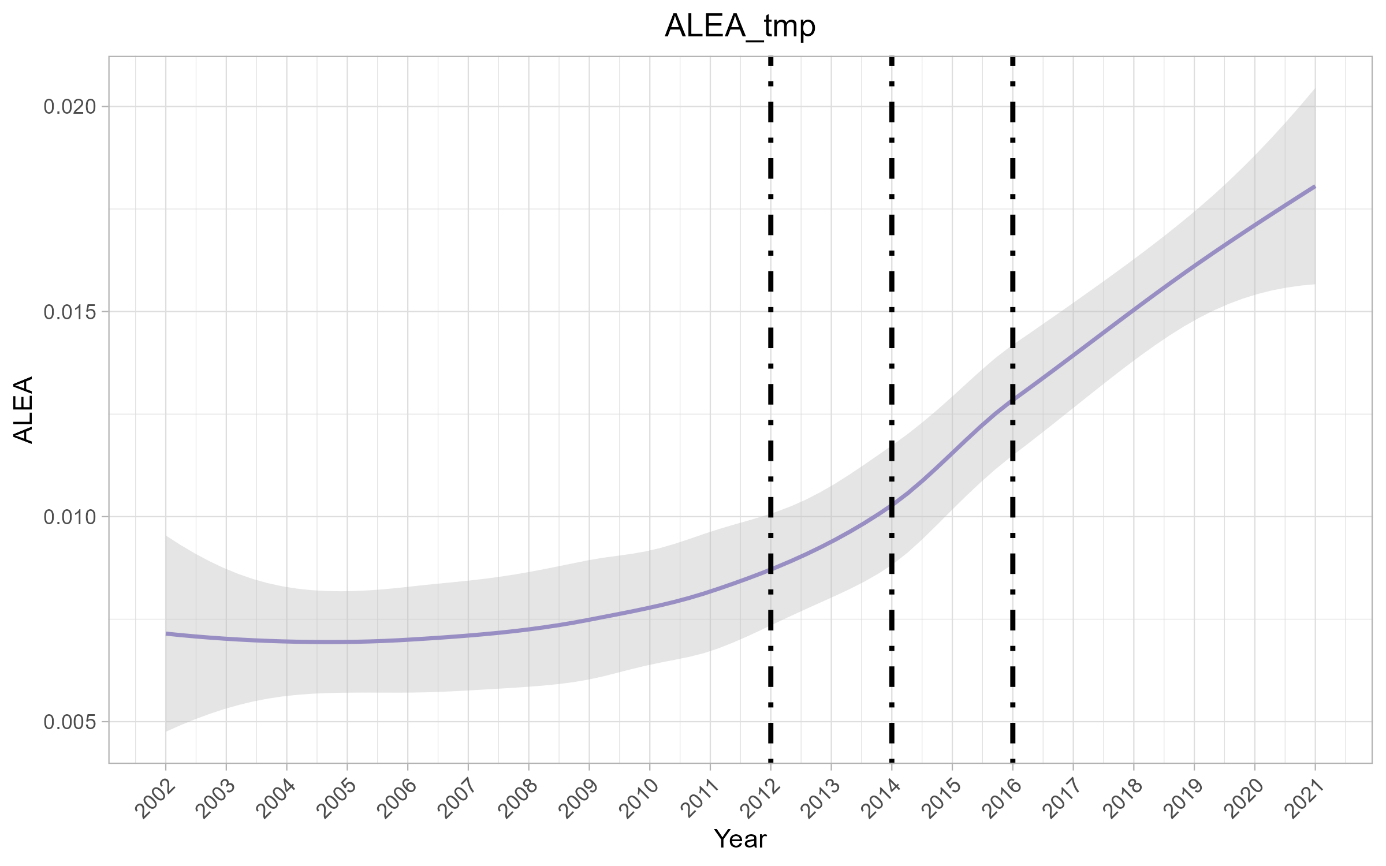


**Figure S8.** Trends of trimethoprim and sulphonamides use in cattle over 2002-2021, expressed as ALEA. Source: Anses 2022^9^

ALEA; animal level of exposure to antimicrobials. Grey zone around the curve represents the 95% confidence interval. Dashed black vertical lines represent the years of implementation of national policy interventions (namely 2012 for Ecoantibio1, 2014 for the national law on the future of agriculture, food and forestry, and 2016 for the decree n° 2016-317 on 3GC-4GC and fluoroquinolones)


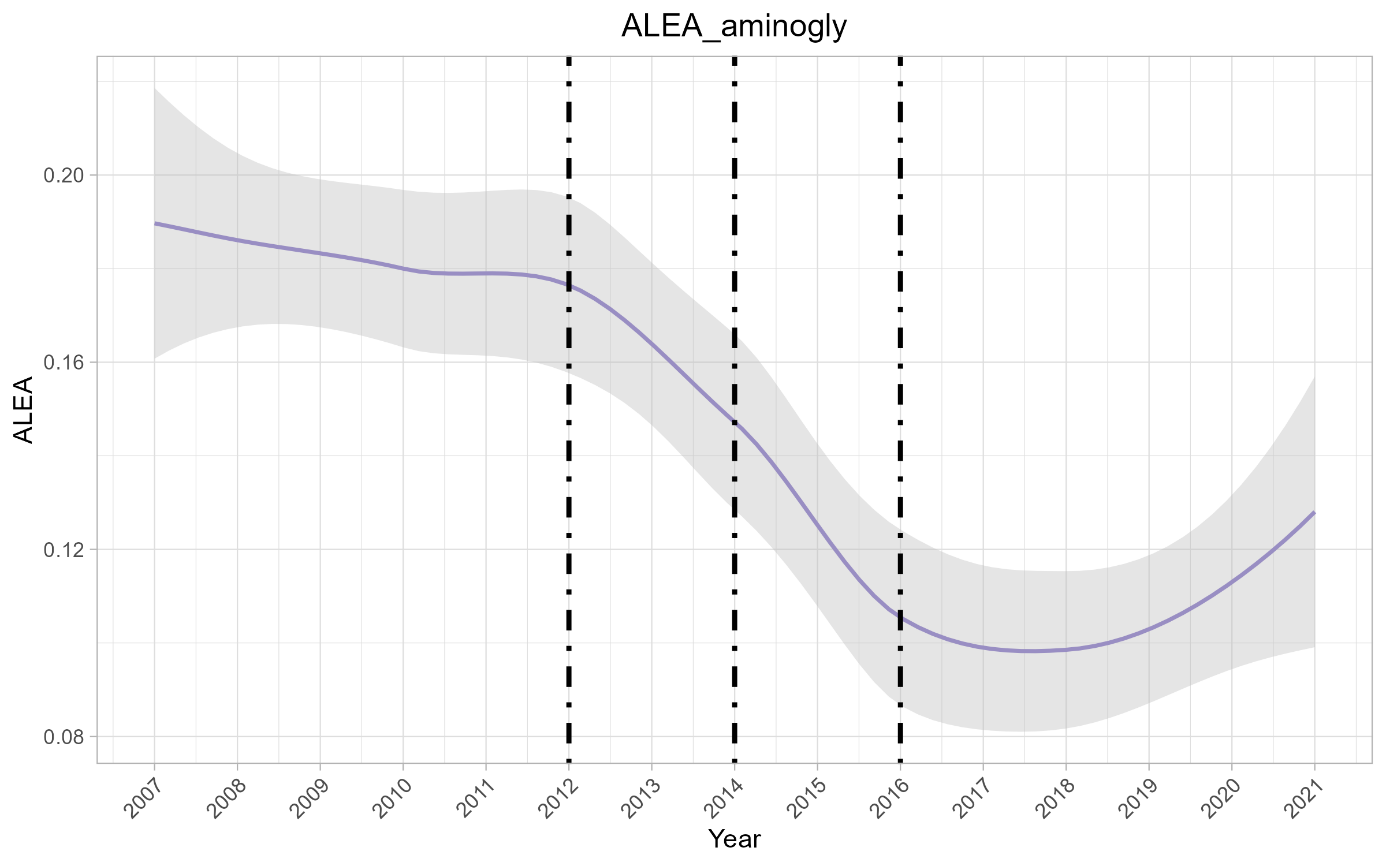


**Figure S9**. Trends of aminoglycosides use in dogs and cats over 2007-2021, expressed as ALEA. Source: Anses 2022^9^

ALEA; animal level of exposure to antimicrobials. Grey zone around the curve represents the 95% confidence interval. Dashed black vertical lines represent the years of implementation of national policy interventions (namely 2012 for Ecoantibio1, 2014 for the national law on the future of agriculture, food and forestry, and 2016 for the decree n° 2016-317 on 3GC-4GC and fluoroquinolones)


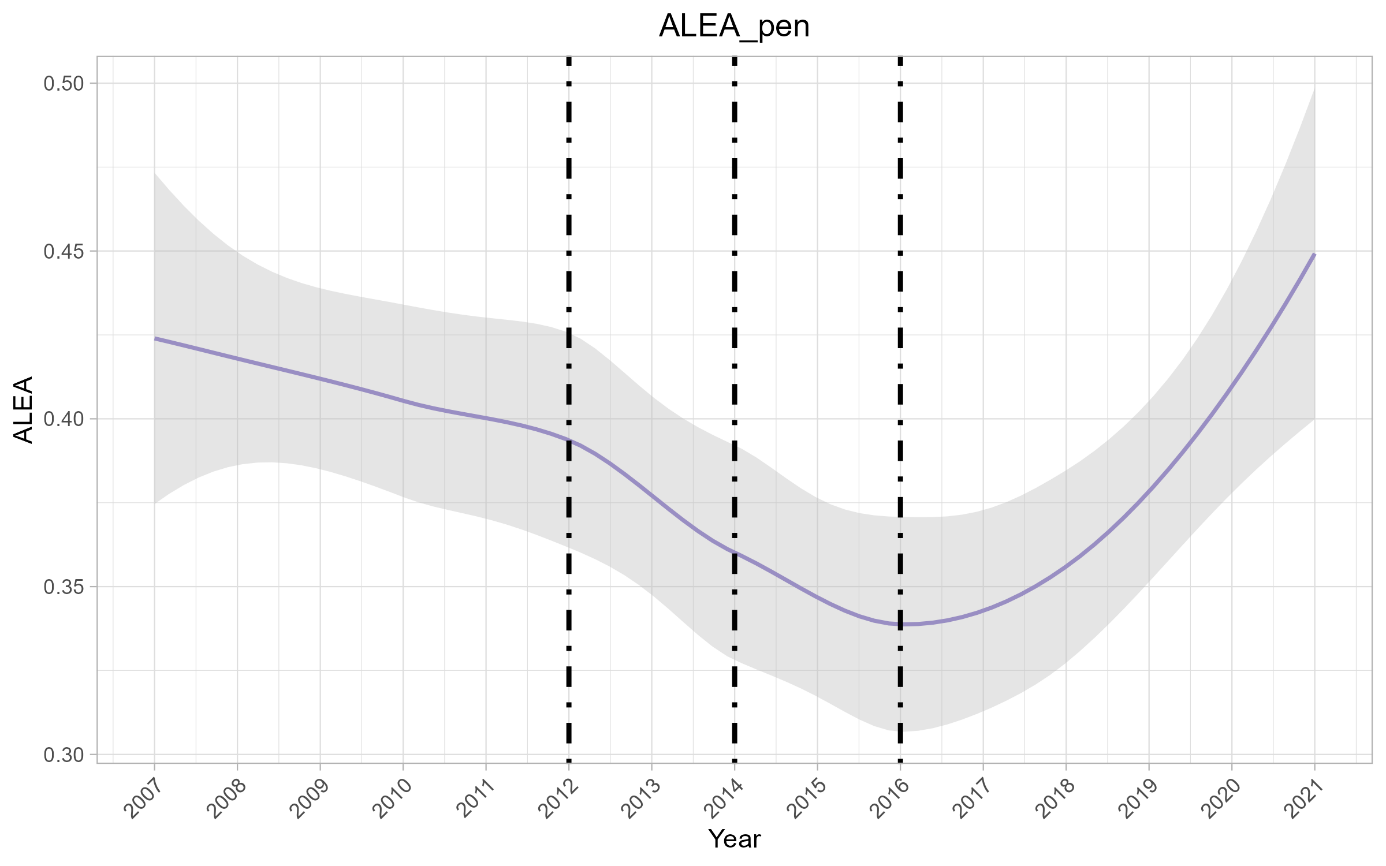


**Figure S10.** Trends of penicillins use in dogs and cats over 2007-2021, expressed as ALEA. Source: Anses 2022^9^

ALEA; animal level of exposure to antimicrobials. Grey zone around the curve represents the 95% confidence interval. Dashed black vertical lines represent the years of implementation of national policy interventions (namely 2012 for Ecoantibio1, 2014 for the national law on the future of agriculture, food and forestry, and 2016 for the decree n° 2016-317 on 3GC-4GC and fluoroquinolones)


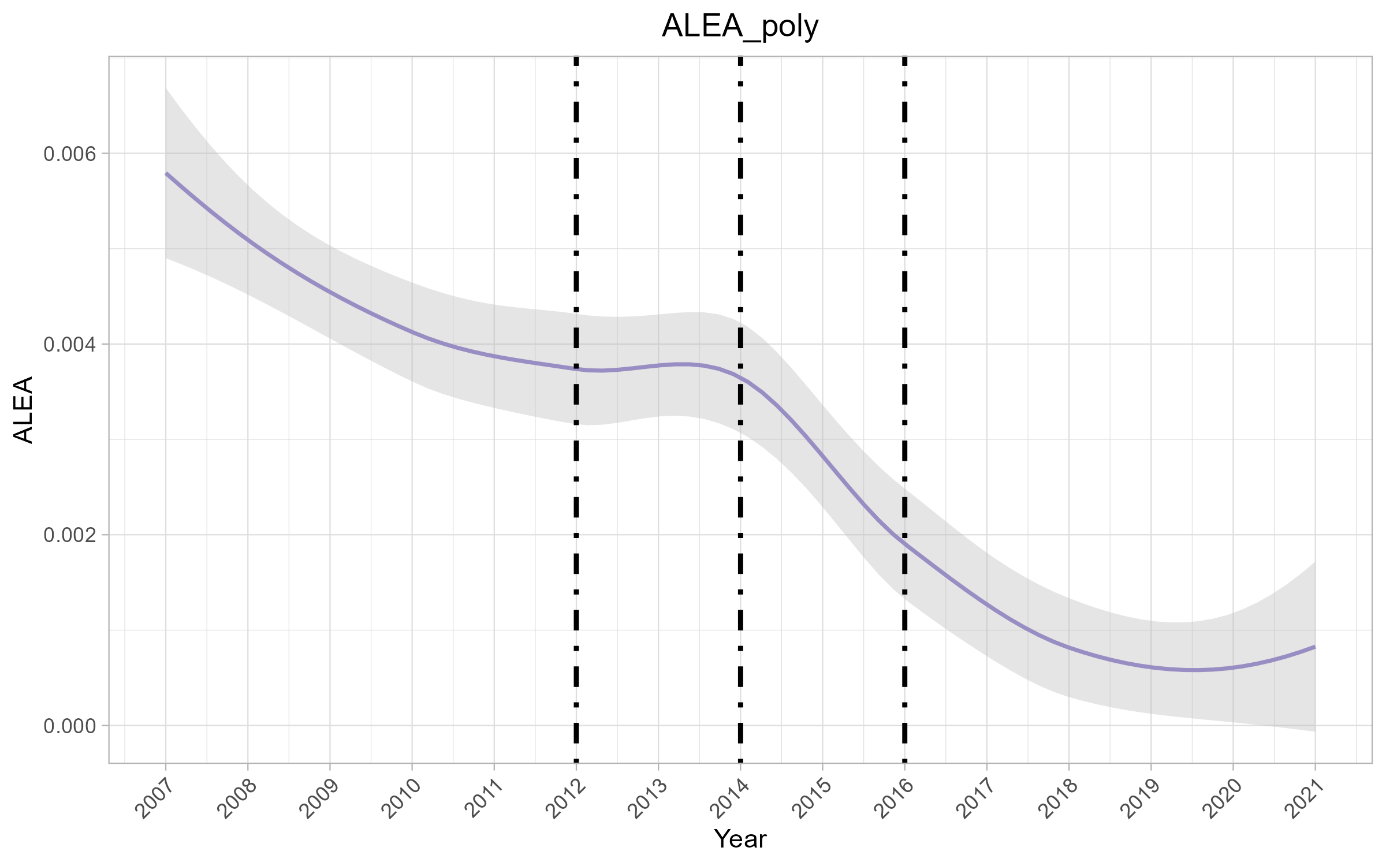


**Figure S11**. Trends of polymyxins use in dogs and cats over 2007-2021, expressed as ALEA. Source: Anses 2022^9^

ALEA; animal level of exposure to antimicrobials. Grey zone around the curve represents the 95% confidence interval. Dashed black vertical lines represent the years of implementation of national policy interventions (namely 2012 for Ecoantibio1, 2014 for the national law on the future of agriculture, food and forestry, and 2016 for the decree n° 2016-317 on 3GC-4GC and fluoroquinolones)


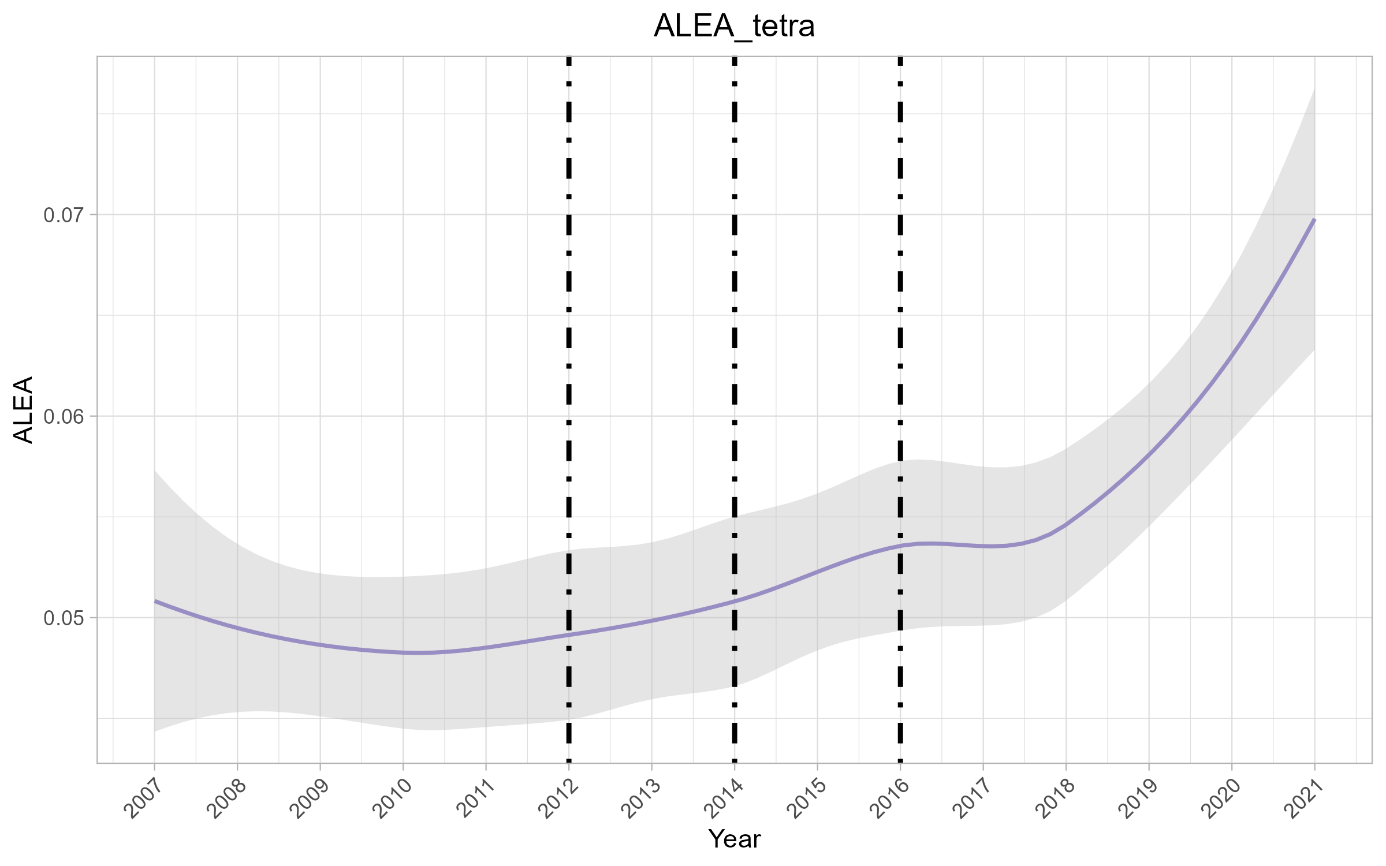


**Figure S12.** Trends of tetracyclines use in dogs and cats over 2007-2021, expressed as ALEA. Source: Anses 2022^9^

ALEA; animal level of exposure to antimicrobials. Grey zone around the curve represents the 95% confidence interval. Dashed black vertical lines represent the years of implementation of national policy interventions (namely 2012 for Ecoantibio1, 2014 for the national law on the future of agriculture, food and forestry, and 2016 for the decree n° 2016-317 on 3GC-4GC and fluoroquinolones)


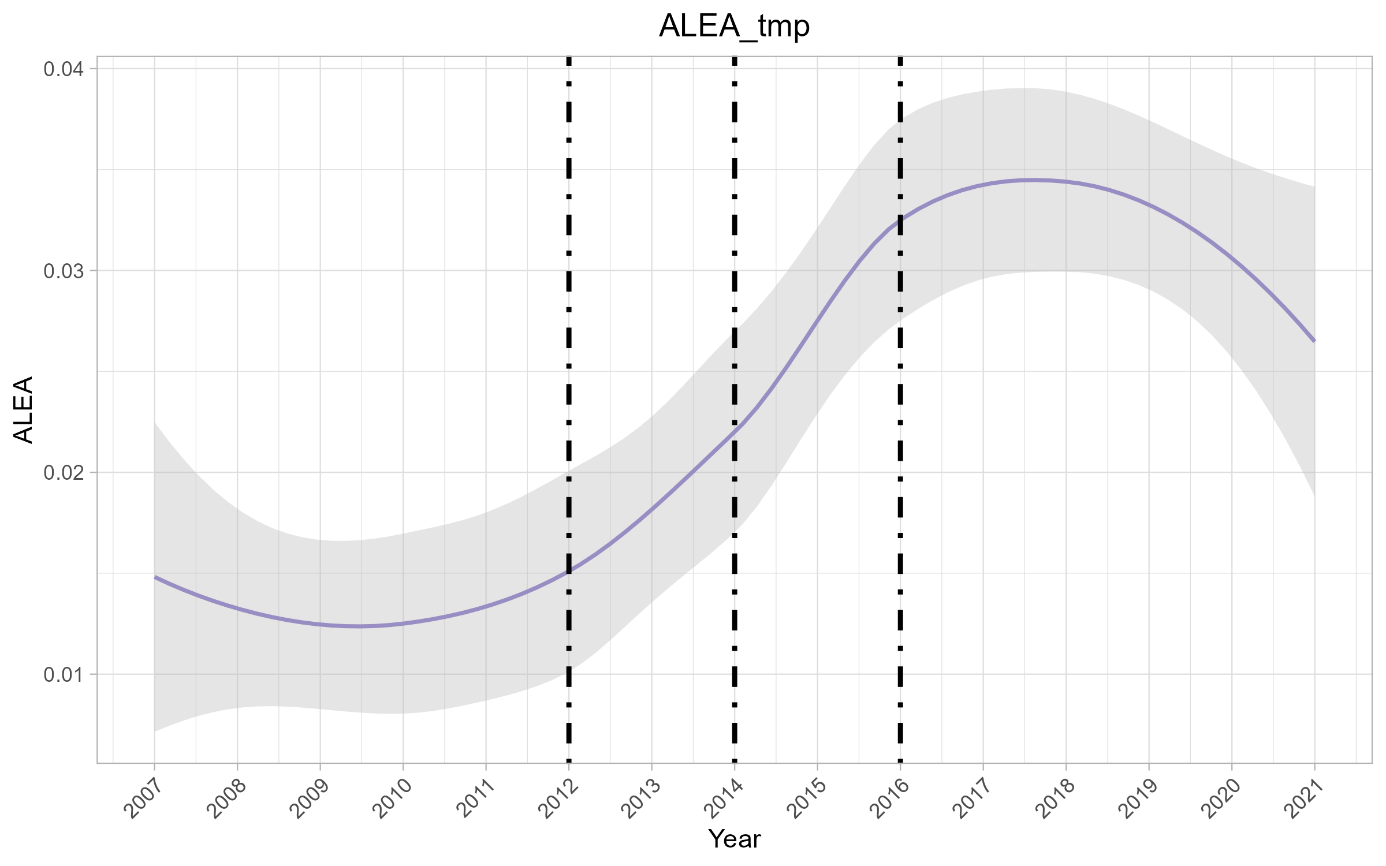


**Figure S13**. Trends of trimethoprim - sulfonamides use in dogs and cats over 2007-2021, expressed as ALEA. Source: Anses 2022^9^

ALEA; animal level of exposure to antimicrobials. Grey zone around the curve represents the 95% confidence interval. Dashed black vertical lines represent the years of implementation of national policy interventions (namely 2012 for Ecoantibio1, 2014 for the national law on the future of agriculture, food and forestry, and 2016 for the decree n° 2016-317 on 3GC-4GC and fluoroquinolones)


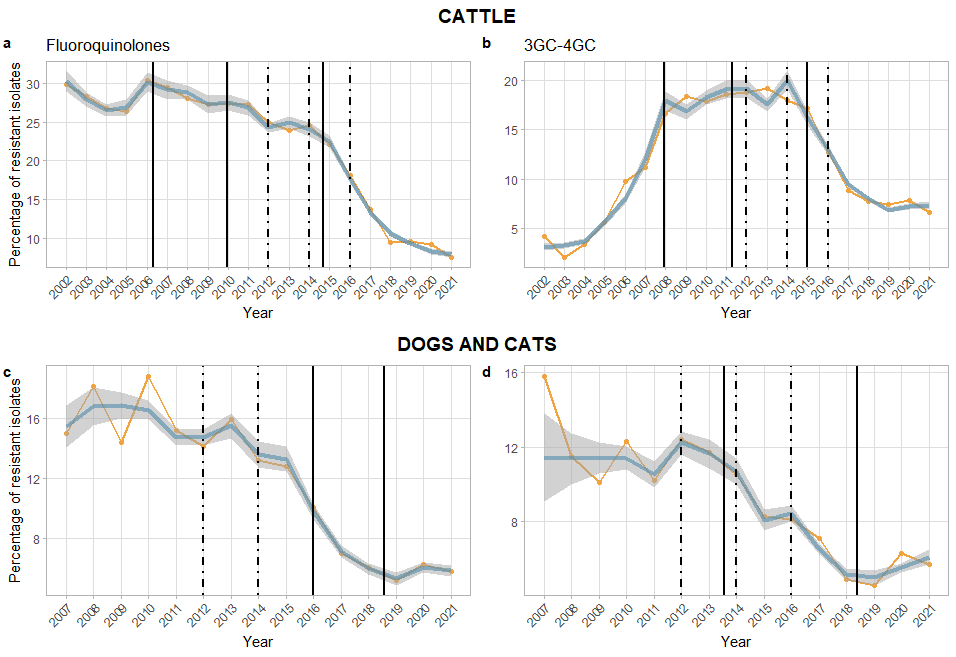


**Figure S14.** Breaking time points as estimated by the segmented regression model for S14a: *E. coli* resistant to fluoroquinolones in young cattle; S14b: *E. coli* resistant to third- and fourth-generation cephalosporins (3GC-4GC) in young cattle; S14c: *E. coli* resistant to fluoroquinolones in dogs and cats; S14d: *E. coli* resistant to 3GC-4GC in dogs and cats.

The yellow curve represents the observed resistance percentage, and the blue curve represents the resistance percentage as predicted by the multivariate regression model together with its 95% confidence interval (grey area around the curve). The dashed black vertical lines represent the years of implementation of national policy interventions (namely 2012 for Ecoantibio1, 2014 for the national law on the future of agriculture, food and forestry, and 2016 for the decree n° 2016-317 on 3GC-4GC and fluoroquinolones). The solid black vertical lines represent the breaking time points estimated by the segmented regression model. Please note that solid and dashed vertical lines overlap in 2016 in Fig S14c. Confidence intervals around the breaking time points are presented in supplementary material (Tables S2 to S5).

**Table S1.** List of antibiotic agents considered to define resistance to fluoroquinolones and third- and-fourth generation cephalosporins in this study

| Antibiotic class | Antibiotic agent tested |
| --- | --- |
| Fluoroquinolones | Ciprofloxacin  Danofloxacin  Difloxacin  Enrofloxacin  Marbofloxacin  Pradofloxacin |
| Third- and fourth-generation cephalosporins | Cefepim  Cefixime  Cefoperazone  Cefotaxim  Cefovecin  Ceftazidime  Ceftiofur  Cefquinome |

Resistance to the antibiotic class was assumed in case resistance to at least one of the molecule was reported. The categories I and R were grouped together into a ‘resistant’ category.

**Table S2.** Confidence intervals around the breaking time points estimated for *E. coli* resistant to fluoroquinolones in young cattle

|  | Estimated breakpoint | Lower bound of the 95% confidence interval | Higher bound of the 95% confidence interval |
| --- | --- | --- | --- |
| Breakpoint #1 | 2006.31 | 2005.68 | 2006.95 |
| Breakpoint #2 | 2009.97 | 2008.57 | 2011.37 |
| Breakpoint #3 | 2014.69 | 2013.71 | 2015.67 |

**Table S3.** Confidence intervals around the breaking time points estimated for *E. coli* resistant to 3GC-4GC in young cattle

|  | Estimated breakpoint | Lower bound of the 95% confidence interval | Higher bound of the 95% confidence interval |
| --- | --- | --- | --- |
| Breakpoint #1 | 2007.95 | 2007.52 | 2008.38 |
| Breakpoint #2 | 2011.29 | 2010.74 | 2011.84 |
| Breakpoint #3 | 2015 | 2014.33 | 2015.67 |

**Table S4.** Confidence intervals around the breaking time points estimated for *E. coli* resistant to fluoroquinolones in dogs and cats

|  | Estimated breakpoint | Lower bound of the 95% confidence interval | Higher bound of the 95% confidence interval |
| --- | --- | --- | --- |
| Breakpoint #1 | 2016 | 2014.93 | 2017.07 |
| Breakpoint #2 | 2018.57 | 2018.07 | 2019.06 |

**Table S5.** Confidence intervals around the breaking time points estimated for *E. coli* resistant to 3GC-4GC in dogs and cats

|  | Estimated breakpoint | Lower bound of the 95% confidence interval | Higher bound of the 95% confidence interval |
| --- | --- | --- | --- |
| Breakpoint #1 | 2013.57 | 2012.9 | 2014.24 |
| Breakpoint #2 | 2018.4 | 2017.83 | 2018.98 |
